# Evaluation of the contribution of the transmembrane region to the ectodomain conformation of the human immunodeficiency virus (HIV-1) envelope glycoprotein

**DOI:** 10.1186/s12985-017-0704-x

**Published:** 2017-02-16

**Authors:** Hanh T. Nguyen, Navid Madani, Haitao Ding, Emerald Elder, Amy Princiotto, Christopher Gu, Patrice Darby, James Alin, Alon Herschhorn, John C. Kappes, Youdong Mao, Joseph G. Sodroski

**Affiliations:** 1 Department of Cancer Immunology and Virology, Dana-Farber Cancer Institute, Department of Microbiology and Immunobiology, Harvard Medical School, 450 Brookline Avenue, CLS 1010, Boston, MA 02215 USA; 2 Department of Medicine, University of Alabama at Birmingham, Birmingham, AL 35294 USA; 3 Birmingham Veterans Affairs Medical Center, Research Service, Birmingham, AL 35233 USA; 4 Department of Immunology and Infectious Diseases, Harvard T. H. Chan School of Public Health, Boston, MA 02215 USA

**Keywords:** HIV-1 Env, gp41, Transmembrane region, Stabilize, Ectodomain, Fibritin –Trimer

## Abstract

**Background:**

The human immunodeficiency virus (HIV-1) envelope glycoprotein (Env), a Type 1 transmembrane protein, assembles into a trimeric spike complex that mediates virus entry into host cells. The high potential energy of the metastable, unliganded Env trimer is maintained by multiple non-covalent contacts among the gp120 exterior and gp41 transmembrane Env subunits. Structural studies suggest that the gp41 transmembrane region forms a left-handed coiled coil that contributes to the Env trimer interprotomer contacts. Here we evaluate the contribution of the gp41 transmembrane region to the folding and stability of Env trimers.

**Methods:**

Multiple polar/charged amino acid residues, which hypothetically disrupt the stop-transfer signal, were introduced in the proposed lipid-interactive face of the transmembrane coiled coil, allowing release of soluble cleavage-negative Envs containing the modified transmembrane region (TM_mod_). We also examined effects of cleavage, the cytoplasmic tail and a C-terminal fibritin trimerization (FT) motif on oligomerization, antigenicity and functionality of soluble and membrane-bound Envs.

**Results:**

The introduction of polar/charged amino acids into the transmembrane region resulted in the secretion of soluble Envs from the cell. However, these TM_mod_ Envs primarily formed dimers. By contrast, control cleavage-negative sgp140 Envs lacking the transmembrane region formed soluble trimers, dimers and monomers. TM_mod_ and sgp140 trimers were stabilized by the addition of a C-terminal FT sequence, but still exhibited carbohydrate and antigenic signatures of a flexible ectodomain structure. On the other hand, detergent-solubilized cleaved and uncleaved Envs isolated from the membranes of expressing cells exhibited "tighter” ectodomain structures, based on carbohydrate modifications. These trimers were found to be unstable in detergent solutions, but could be stabilized by the addition of a C-terminal FT moiety. The C-terminal FT domain decreased Env cleavage and syncytium-forming ability by approximately three-fold; alteration of the FT trimerization interface restored Env cleavage and syncytium formation to near-wild-type levels.

**Conclusion:**

The modified transmembrane region was not conducive to trimerization of soluble Envs. However, for HIV-1 Env ectodomains that are minimally modified, membrane-anchored Envs exhibit the most native structures and can be stabilized by appropriately positioned FT domains.

## Background

Human immunodeficiency virus (HIV-1) entry into cells is mediated by the envelope glycoprotein (Env) spike on the viral membrane [1]. The trimeric Env complex is a Type 1 membrane protein composed of three gp120 exterior Env and three gp41 transmembrane Env subunits [1–3]. Synthesized in the rough endoplasmic reticulum, the ~850-residue Env precursor is cotranslationally modified by high-mannose N-linked glycans, is anchored in the membrane by a transmembrane region, and trimerizes [4–8]. Env is then transported to the Golgi apparatus, where accessible high-mannose glycans are processed to complex carbohydrates [8–10]. The glycosylated gp160 Env precursor is then cleaved into the mature gp120 and gp41 Envs in the Golgi compartment, just prior to Env transport to the surface of the infected cell and incorporation into virions [10, 11]. The unliganded Env spike on the HIV-1 membrane exists in a high-potential-energy state (State 1) [12–14]. Binding of gp120 to the initial target cell receptor, CD4, induces conformational changes in the metastable Env complex that lead to lower-energy intermediate states (States 2 and 3) along the entry pathway [13–16]. Env-CD4 binding allows gp120 to bind the coreceptor, either CCR5 or CXCR4 [17–24], and induces the pre-hairpin intermediate (State 3), in which three gp41 helices (the heptad repeat 1 (HR1) regions) form an extended coiled coil near the trimer axis [25–28]. The hydrophobic “fusion peptide” at the gp41 amino terminus is directed towards the target cell membrane as a result. The binding of gp120 to the coreceptor leads to the conversion of the pre-hairpin intermediate to an energetically stable six-helix bundle. Because of the interaction of the gp41 fusion peptide with the target cell membrane and the anchorage of the gp41 transmembrane region in the viral membrane, six-helix bundle formation approximates and fuses these membranes [29–33]. Thus, transitions from the high potential energy state of the unliganded Env trimer drive fusion between the viral and cell membranes.

HIV-1 Env ectodomains have been produced as soluble glycoproteins for structural studies and for use as immunogens [34–43]. The lability of these soluble oligomers suggests that the interprotomer contacts are weak, and has led to various efforts to produce more stable Env trimers. These include: 1) the addition of heterologous trimerization motifs at the C-termini of uncleaved Env ectodomains [41, 44–47]; 2) the introduction of the I559P change in gp41, mutation of residues 501 and 605 to cysteine to form an SOS disulfide bond linking the gp120 and gp41 subunits of cleaved Env, and truncation of the gp41 ectodomain at residue 664 (the SOSIP.664 modifications) [48–57]; and 3) the addition of flexible linkers at the gp120-gp41 cleavage site of soluble gp140 SOSIP.664 trimers [58, 59]. In all of these cases, the gp41 transmembrane region has been deleted to produce soluble Env constructs. In a recent cryo-electron microscopy (cryo-EM) structure of a membrane HIV-1 Env complexed with a neutralizing antibody Fab fragment, the membrane-proximal and transmembrane regions were disordered and not resolved [60]. However, a cryo-EM study of an unliganded, uncleaved membrane HIV-1 Env (Env(−)Δ712) solubilized in detergent suggested that the transmembrane helices form a left-handed trimeric coiled coil [61, 62]. The transmembrane region of immunodeficiency viruses is longer (~22 amino acid residues) than that of other retroviruses, and contains a basic residue, Arg 696, near the middle of the membrane-spanning region [63–65]. A recent NMR structure of the HIV-1 gp41 transmembrane peptide in detergent-lipid bicelles confirmed the potential of this region to form a left-handed coiled coil, with a central kink near the basic arginine residue at position 696 [66]. Near the C-terminus of the transmembrane region, a hydrophilic core composed of polar and charged residues stabilized the structure.

A number of observations suggest that the gp41 transmembrane region and membrane-proximal external region (MPER) make important contributions to the structure of the HIV-1 Env ectodomain [12, 66–78]. Changes in the HIV-1 transmembrane region have been shown to decrease the efficiency of virus entry and cell-cell fusion, even when the Env mutants were efficiently expressed and incorporated into virions [67–70]. In one such mutant, the arginine residue at position 696 was converted to a hydrophobic residue, completely eliminating Env function [70]. In addition, significant changes in the hydrophilic core of the transmembrane region can alter HIV-1 sensitivity to neutralization by antibodies [66]. Similarly, changes in the gp41 MPER have been shown to influence HIV-1 susceptibility to antibody or small-molecule inhibition [71–78]. For example, alteration of the well-conserved hydrophobic MPER residue, Trp 680, resulted in a significant increase in the sensitivity of HIV-1 to neutralizing antibodies and small-molecule CD4-mimetic compounds [74]. Apparently, the membrane-interactive regions of gp41 help to maintain the native unliganded state (State 1) of the HIV-1 Env trimer.

The above findings suggest that HIV-1 Env constructs with missing, disordered or aberrant transmembrane regions or MPERs might sample ectodomain conformations other than the native unliganded state. Here we evaluate different strategies for including the gp41 transmembrane region in HIV-1 Env constructs, paying particular attention to the effects of such manipulation on Env trimer's stability, glycosylation and antigenicity.

## Methods

### Envelope glycoprotein constructs

All of the glycoproteins used in this study were derived from the HIV-1_JR-FL_ Env with a truncation (Δ712) of the cytoplasmic tail, unless otherwise noted [61, 62]. HIV-1 *env* cDNA was codon-optimized and subcloned into the pcDNA3.1(−) expression plasmid (Invitrogen) using 5’ Xba I and 3’ Afl II sites. Env cleavage was abolished by the R508S + R511S changes. All Env amino acid residues are numbered by alignment with the prototypic HXBc2 sequence, according to current convention [79]. Each of the TM_mod_1-17 glycoproteins has six changes in the gp41 transmembrane region involving residues I688, L692, L695, V698, L702 and V705. The TM_mod_18 glycoprotein is altered at residues I686, V693, L697 and T700. The soluble sgp140(−) glycoprotein was produced from an expressor plasmid in which the sequence encoding the transmembrane region of HIV-1_JR-FL_ Env(−)Δ712 was deleted. TM_mod_10v2 is identical to the TM_mod_10 glycoprotein except for three additional changes: M687D, L697A and F699A. TMmod10v3 is identical to TMmod10v2 except that the residues at the e and g positions (L692, L697 and F699) are wild-type in sequence. All primers for mutagenesis were designed using the online Agilent Technologies Quikchange Primer Design program. These mutations were introduced by site-directed mutagenesis PCR using Pfu Ultra II polymerase (Agilent Technologies), following the manufacturer’s protocol. For some constructs, the E168K + N188A changes in the gp120 V2 region were also added to allow HIV-1_JR-FL_ Env recognition by the PG9 and PG16 antibodies.

In the TM_mod_10modCS Env mutant, the R_508_EKR cleavage site in TM_mod_10 was replaced by a flexible linker (GGS)_4_. The linker was inserted using overlap extension PCR. The insert was cloned from two fragments: the 5’ fragment starts before the Bsr GI site and covers the new linker: RDNWRSELYKYKVVKIEPLGVAPTKAKRRVVQGGSGGSGGSGGSAVGIGAV. The 3’ fragment encodes the part of the linker beginning at A512 and ends after the Afl II insertion site. The longer overlapped fragment was cloned using appropriate primers, and the insert was digested and cloned into the *env* expressor plasmid using the Bsr GI and Afl II sites.

To introduce the fibritin (FT) trimerization motif [80], a short (GGSG)_2_ linker followed by the fibritin sequence (GYIPEAPRDGQAYVRKDGEWVLLSTFL) was added to the C-termini of the soluble envelope constructs (sgp140 and TM_mod_10) and the membrane-anchored envelope constructs (Env(−)Δ712 and Env(+)Δ712). To disrupt trimerization of the fibritin domain, the Y469A and R471A changes (fibritin E protein numbering) were introduced into the Env(+)Δ712 construct to produce Env(+)Δ712 FT_mut_. TM_mod_1-18 and sgp140(−) Envs were tagged with His_6_. TM_mod_10 EKNA, TM_mod_10 (+) EKNA, TM_mod_10 modCS EKNA and TM_mod_10 EKNA Envs with different cytoplasmic tails are Strep-tagged. TM_mod_10v2 Env was not tagged and was compared to the untagged TM_mod_10 Env. All Envs used in the fibritin experiments (Figs. 4 and 5) are His_6_ tagged.

### Cell lines

293T cells (ATCC) were grown in Dulbecco’s modified Eagle’s medium supplemented with 10% fetal bovine serum (FBS) and 100 μg/ml of penicillin-streptomycin (Life Technologies). Cf2Th cells (ATCC) stably expressing human CD4 and CCR5 were grown in the same medium additionally supplemented with 0.4 mg/ml of G418 and 0.2 mg/ml of hygromycin (Life Technologies).

### Expression and oligomerization of soluble envelope glycoproteins

293T cells were transiently transfected with plasmids expressing soluble Envs using polyethylenimine following a standard protocol. Forty-eight to seventy-two hours post-transfection, cellular supernatants and lysates were collected, clarified and analyzed by reducing SDS-PAGE. To test for protein oligomerization, supernatants were analyzed by Blue Native PAGE following ThermoFisher’s protocol. Working dilutions for Western blotting were 1:2,000 goat anti-gp120 polyclonal antibody (ThermoFisher), 1:2,000 4E10 anti-gp41 antibody (Polymun Scientific, NIH AIDS Reagent Program), 1:10,000 mouse anti-β- actin (Abcam), 1:3,000 HRP-conjugated goat anti-human IgG (SantaCruz), 1:3,000 HRP-conjugated rabbit anti-goat IgG (ThermoFisher), and 1:10,000 HRP-conjugated goat anti-mouse IgG (ThermoScientific).

### Deglycosylation of Env glycoproteins

Supernatants containing soluble glycoproteins or envelope proteins purified from cell membranes were denatured and treated with PNGase F or Endo H_f_ enzymes (New England BioLabs) for 1 ½ hours following the manufacturer’s protocol. The deglycosylated glycoproteins were then subjected to reducing SDS-PAGE as described. For glycoprotein treated with kifunensine (R&D Systems), transfected cells transiently expressing Envs were continuously incubated in 50 mM kifunensine before cellular supernatants were collected, clarified and deglycosylated as described.

### Immunoprecipitation of soluble glycoproteins

For immunoprecipitation, 100 μL of cellular supernatants containing soluble glycoproteins was incubated with 25 μl Protein A-agarose beads (Sigma Aldrich) that had been hydrated and resuspended to 100 μg/ μl concentration in IP buffer (20 mM Tris–HCl (pH 8.0), 300 mM NaCl supplemented with 0.1% NP-40). HIV-1 broadly neutralizing antibodies (2G12, VRC01, PG9, PG16) and weakly neutralizing antibodies (19b, 17b) at 10 μg/ml ﻿were ﻿added ﻿and samples were incubated for 2 h at room temperature. The 17b antibody was also incubated with Envs in presence of soluble CD4 at 20 μg/ml. Beads were washed three times with IP buffer/NP-40 and once in IP buffer. Beads were then resuspended in 35 μL total volume of IP buffer supplemented with 1X NuPAGE LDS Sample Buffer and 100 mM dithiothreitol (DTT). Samples were boiled and supernatants were analyzed by SDS-PAGE and blotted with goat anti-gp120 polyclonal antibody and HRP-conjugated rabbit anti-goat IgG.

### Immunoprecipitation of cell-surface Envs

293T cells were transfected with plasmids expressing the HIV-1_JR-FL_ Env(−)Δ712, Env(−)Δ712 FT, Env(+)Δ712 and Env(+)Δ712 FT glycoproteins, using polyethylenimine. Seventy-two hours after transfection, cells were washed and resuspended in PBS/ 5% FBS. Cells were incubated with 10 μg/ml antibodies for one hour at room temperature and then washed once in PBS. Cells were then lysed and the lysates were incubated overnight at 4 °C with Protein A-Sepharose beads prepared as described above. The precipitates were washed with IP buffer and analyzed by Western blotting, as described above.

### Assessing proteolytic cleavage of cell-surface Envs

293T cells in 6-well plates were transfected with plasmids expressing the HIV-1 Env(+)Δ712, Env(+)Δ712 FT and Env(+)Δ712 FT_mut_ glycoproteins, at 0.5 μg DNA per well, using Effectene (Qiagen) according to the manufacturer’s protocol. Approximately 40 h after transfection, cell-surface Env glycoproteins were biotinylated using the Cell Surface Protein Isolation Kit (Thermo Scientific), according to the manufacturer’s protocol. Briefly, cells were washed twice in ice-cold PBS, scraped, resuspended in biotin solution, and incubated at 4 °C for 30 min. Cells were then washed and lysed. Clarified lysates were incubated with NeutrAvidin beads for one hour at room temperature. Beads were washed, resuspended in IP buffer supplemented with 1X NuPAGE LDS Sample Buffer and 100 mM DTT, and boiled for 10 min (100 °C). The denatured glycoproteins were then treated with PNGase F as described above. The digested glycoproteins were Western blotted with the 4E10 antibody.

### Alpha-complementation assay

Cf2Th-CD4/CCR5 cells plated in 96-well plates were transfected with the ω-gal-expressing plasmid using Effectene reagent (Qiagen). 293T cells plated in 6-well plates were transfected with plasmids expressing α-gal and Env at a 1:1 ratio using the Effectene reagent (2 μg total DNA/well). Seventy-two hours after transfection, the 293T cells were briefly trypsinized, washed and added to the ω-gal-expressing Cf2Th-CD4/CCR5 cells (one well of 293T cells is sufficient for 12 wells of Cf2Th-CD4/CCR5 cells). Cells were incubated at 37 °C/5% CO_2_ for 6 h before they were washed and lysed in 30 μL/well Galacto-Star lysis buffer (Applied Biosciences). Galacto-Star substrate (diluted 1:50 in 100 μL/well Galacto-Star buffer diluent) was then added, and plates were incubated at room temperature for 1 h before signal was measured in an EG&G Berthold LB 96 V microplate luminometer at 1-s intervals.

### Flow cytometry

Seventy-two hours after transfection, 293T cells were washed, briefly trypsinized and resuspended in PBS/5% FBS buffer. Cells were incubated with 10 μg/mL 2G12 or VRC01 antibody for 30 min at room temperature. Cells were then washed once using the same buffer before incubating with allophycocyanin (APC)-conjugated anti-human IgG (Jackson ImmunoResearch Laboratories) at an 1:100 dilution for 15 min at room temperature. Cells were washed, resuspended in PBS/FBS buffer and analyzed by a BD FACS Canto instrument. Geometric means of the APC signal were used for analysis.

### Purification of membrane Env proteins

293T cells were transfected with plasmids encoding Env(−)Δ712, Env(−)Δ712 FT, Env(+)Δ712 and Env(+)Δ712 FT glycoproteins. Forty-eight to seventy-two hours later, cells were washed, trypsinized briefly and pelleted at 4 °C. All subsequent steps were performed at 4 °C. Pellets were resuspended in five volumes of homogenization buffer (250 mM sucrose, 10 mM Tris–HCl (pH 7.4), 1 mM EDTA, 1X protease inhibitor) and homogenized in a glass Dounce homogenizer with 100 strokes. Homogenates were centrifuged at 1000 × *g* for 10 min. Supernatants were collected and centrifuged at 10,000 × *g* for 10 min. Supernatants were again collected and centrifuged in a fixed-angle rotor at 100,000 × *g* for 30 min. Supernatants were aspirated and the pellets were resuspended in resuspension buffer (20 mM Tris–HCl (pH 7.4), 300 mM NaCl, 100 mM (NH_4_)_2_SO_4_, 0.02% sodium azide, 1X protease inhibitor) and homogenized in a small Dounce homogenizer on ice with 100 strokes. Samples were centrifuged at 100,000 × *g* for 45 min. Supernatants were aspirated and the pellets were resuspended in solubilization buffer (100 mM (NH_4_)_2_SO_4_, 20 mM Tris–HCl (pH 7.5), 150 mM NaCl, 1% Cymal-5, 1X protease inhibitor). The membranes containing Env glycoproteins were lysed by incubation at 4 °C for 30 min on a rocking platform and immediately used for Blue Native PAGE. For Blue Native PAGE analysis, samples were mixed with 0.25% G-250 and 1X NativePAGE Sample Buffer (ThermoFisher), and gels were run following the manufacturer’s protocol for samples with detergents. Samples stored at 4°C were used for deglycosylation studies.

### Generation, purification and infection of pseudotyped viruses

To produce lentiviral virions pseudotyped with Env glycoproteins, 293T cells were co-transfected with the Env-expressing plasmid, the pCMV HIV-1 Gag-Pol packaging construct and the firefly-luciferase-expressing plasmid (at a 1:1:3 weight ratio) following a standard calcium phosphate transfection protocol. Seventy hours after transfection, supernatants containing virions were collected and filtered through 0.45-μm membranes.

To purify and concentrate the pseudotyped virions, 3 mL of the filtered supernatants were layered on 500 μL of a 20% sucrose solution (20 g UltraPure sucrose, 100 mM NaCl, 20 mM HEPES (pH 7.4) and 1 mM EDTA in 100 mL total volume [81]) and centrifuged at 100,000 x g for 1 h at 4 °C. Supernatants were aspirated and pellets were resuspended in 35 μL denaturing buffer (PBS, 1X LDS, 100 mM DTT). Samples were boiled, analyzed by SDS-PAGE, and Western blotted with goat anti-gp120 antibody as described.

To determine the efficiency of infection, filtered cellular supernatants containing virions were added to suspended Cf2Th-CD4/CCR5 cells. The mixture was incubated at 37 °C/5% CO_2_ for 48 h before the cells were lysed in 1X passive lysis buffer (Promega) at 80 μL/well for 24-well plates, freeze-thawed once and cell lysates transferred to black-white 96-well plates. Luciferase activity was then quantified by a luminometer after the addition of 100 μl of luciferin buffer (15 mM MgSO_4_, 15 mM KPO_4_ (pH 7.8), 1 mM ATP, and 1 mM DTT) and 50 μl of 1 mM firefly d-luciferin, potassium salt (Gold Biotechnology). Signals were measured at 20-s intervals by a luminometer.

## Results

### Soluble HIV-1 Envs with modified transmembrane regions

**Fig. 1:**
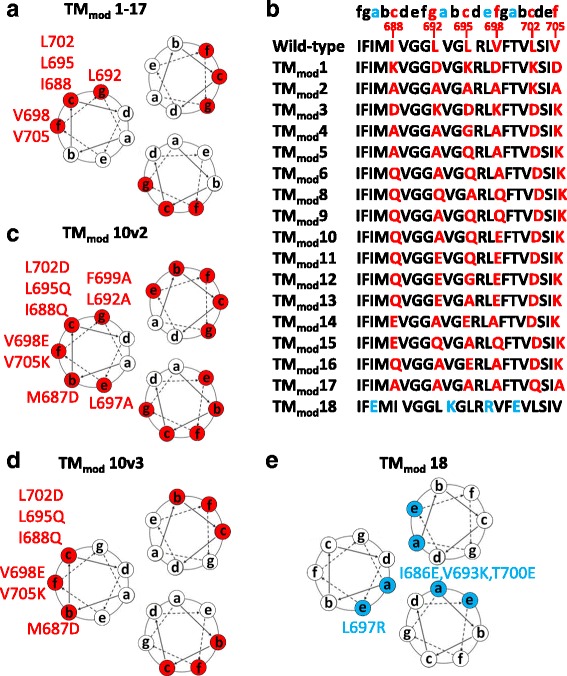
Location of changes in the transmembrane region of HIV-1_JR-FL_ Envs. **a** The projection of the helical coiled coil in the HIV-1 Env transmembrane region, based on the NMR structure of a peptide embedded in bicelles [29], is shown. Six hydrophobic residues (I688, L692, L695, V698, L702 and V705) highlighted in red are predicted to be located on the lipid-interacting surface of the membrane-spanning coiled coil. **b** In the TM_mod_1-17 constructs, the six hydrophobic residues on the putative lipid-interacting surface in **a** were changed to combinations of alanine, glutamine or charged amino acid residues. **c** The TM_mod_10v2 construct is a TM_mod_10 variant with three additional changes (M687D, L697A and F699A) introduced to modify all of the predicted external positions **b**, **c**, **e**, **f**, **g** on the helical coiled coil. **d** The TM_mod_10v3 construct is identical to TM_mod_10v2 except that the residues in the **e** and **g** positions of the coiled coil (L692, L697 and F699) are wild-type in sequence. **e** The TM_mod_18 glycoprotein control contains four changes in the transmembrane region, three of which (I686E, V693K and T700E) are predicted to be located in the interior of the coiled coil (at the **a** position of the heptad repeat sequence)

The HIV-1 gp41 transmembrane region forms a left-handed coiled coil in the cryo-EM structure of the HIV-1_JR-FL_ Env(−)Δ712 trimer and in the NMR structure of a transmembrane region peptide in a detergent-lipid bicelle [62, 66] (Fig. 1a). These structural models predict that six hydrophobic residues are located on the faces of the helical coiled coil that interact with the lipid membrane: Ile 688, Leu 692, Leu 695, Val 698, Leu 702 and Val 705. We hypothesized that alteration of these residues could eliminate the membrane anchorage of the HIV-1_JR-FL_ Env(−)Δ712 glycoprotein while potentially preserving the interprotomer interactions that help maintain trimer conformation. When these residues in the HIV-1_JR-FL_ Env(−)Δ712 glycoprotein were individually altered to alanine, the resulting glycoproteins were efficiently expressed in cells and, like the unmodified Env(−)Δ712 glycoprotein, were completely cell-associated (data not shown). Thus, replacement of these individual non-polar aliphatic residues by alanine did not disrupt the membrane anchorage of the Env(−)Δ712 glycoprotein.

**Fig. 2:**
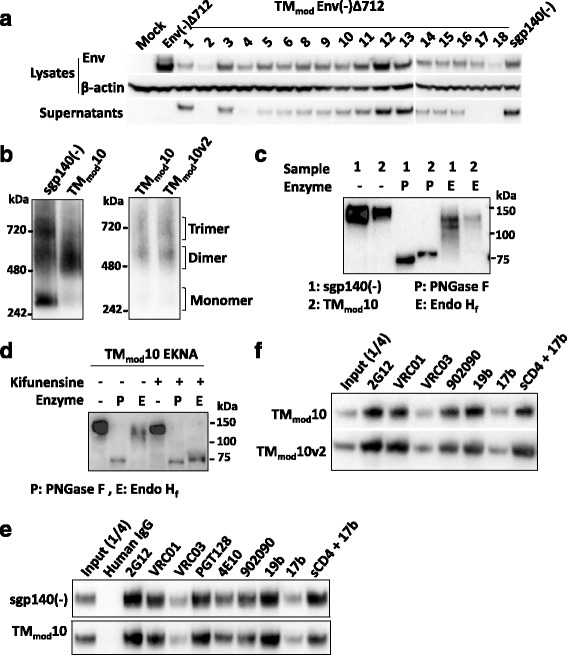
Characterization of TM_mod_ mutant Envs. **a** Cellular lysates and supernatants from 293T cells that were mock-transfected or transfected with TM_mod_ Env DNAs were Western blotted. Western blots shown in this figure used goat anti-gp120 antibody or a mouse anti-β-actin control. The TM_mod_ Envs with at least two new charged residues in the transmembrane region were secreted. The control TM_mod_18 Env was inefficiently expressed and not secreted. The sgp140(−) Env lacks the transmembrane region. **b** The secreted Envs were analyzed by Blue Native PAGE. The sgp140(−) glycoprotein migrated as a heterogeneous mixture of monomers, dimers and trimers. The representative TM_mod_10 protein migrated predominantly as a dimer. The TM_mod_10v2 glycoprotein was also largely dimeric. Note that HIV-1 Envs migrate more slowly than expected in Blue Native gels. **c** Transfected cell supernatants containing sgp140(−) and TM_mod_10 Envs were either mock-treated or treated with PNGase F (which removes all N-linked glycans) or Endo H_f_ (which removes only high-mannose glycans). The Western blot shows that both sgp140(−) and TM_mod_10 Envs resist Endo H_f_ treatment, which indicates that they contain mostly complex carbohydrates. **d** Transfected cells expressing the E168K + N188A (EKNA) variant of TM_mod_10, which allows HIV-1_JR-FL_ Envs to be recognized by the PG9 and PG16 neutralizing antibodies [96–99], were incubated with 50 mM kifunensine (a mannosidase I inhibitor). Cell supernatants were collected, deglycosylated with PNGase F or Endo H_f,_ and Western blotted. Addition of kifunensine converted TM_mod_10 glycosylation from mostly complex glycans to high-mannose glycans. **e, f** Cell supernatants containing the indicated soluble glycoproteins were precipitated with the indicated antibodies, and the precipitates were Western blotted with goat anti-gp120 antibody. The three soluble Envs exhibit a similar pattern of antigenicity. One-fourth volume of the supernatant used for immunoprecipitation was analyzed in the input lane. Data are representative of those obtained in at least two independent experiments

Next, combinations of charged amino acids and glutamine and alanine residues were introduced into all six helical positions predicted to interact with the lipid bilayer (Fig. 1b). Three of the mutants, TM_mod_2, TM_mod_4 and TM_mod_17, were inefficiently expressed and secreted (Fig. 2a). The remaining 13 mutants were secreted into the supernatants of expressing cells (Fig. 2a). Thus, the introduction of at least two charged residues into the transmembrane region appeared to be required for efficient expression and/or secretion of the Env(−)Δ712 glycoprotein.

We also designed a mutant, TM_mod_10v2, in which all of the hydrophobic residues at the b, c, e, f and g positions of the predicted transmembrane helices were altered (Fig. 1c). In the TM_mod_10v3 mutants, the hydrophobic residues at the b, c and f positions of the transmembrane helices were altered (Fig. 1d). The TM_mod_10v2 and TM_mod_10v3 Envs were secreted into the medium of expressing cells (Fig. 2b and data not shown). As a control, four charged amino acid residues were introduced into the Env(−)Δ712 transmembrane region, one into a residue predicted to interact with the lipid bilayer and three located in the interior of the coiled coil (TM_mod_18 in Fig. 1b and e). The TM_mod_18 mutant was inefficiently expressed and did not appear to be secreted from cells (Fig. 2a). Thus, the introduction of multiple charged and polar residues on the helical faces of the transmembrane coiled coil that are predicted to interact with the lipid bilayer resulted in secretion of the Env(−)Δ712 glycoproteins into cell supernatants. These results are consistent with a model in which the surface of the transmembrane helix facing away from the potential trimer interface acts as a stop-transfer signal as Env is inserted into the membrane of the ER.

### Oligomerization state of the TM_mod_ Env(−)Δ712 glycoproteins

**Fig. 3:**
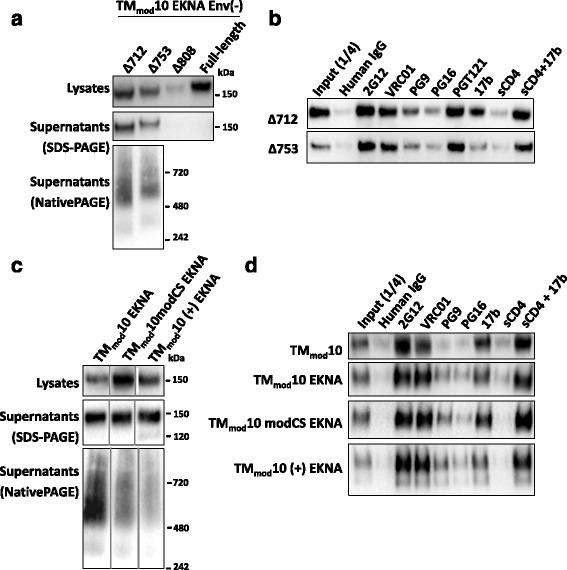
Effects of cytoplasmic tail and cleavage site modifications on TM_mod_10 Env. **a** The TM_mod_10 EKNA variant has the E168K + N188A changes that allow the HIV-1_JR-FL_ Env to be recognized by the PG9 and PG16 antibodies [96–99]. The full-length TM_mod_10 EKNA Env or the TM_mod_10 EKNA variants with a deleted (Δ712) or truncated (Δ753 and Δ808) cytoplasmic tail were expressed in 293T cells. The cell lysates and supernatants were analyzed by SDS-PAGE, and the cell supernatants by Blue Native PAGE. The gels shown in this figure were Western blotted with a polyclonal goat anti-gp120 antibody. Addition of the cytoplasmic tail did not prevent TM_mod_10 EKNA expression in cells, but only the TM_mod_10Δ753 EKNA glycoprotein with the shortest tail was secreted. The secreted TM_mod_10Δ753 Env was mainly dimeric based on the Blue Native gel. **b** The TM_mod_Δ712 and TM_mod_Δ753 EKNA Envs precipitated from the supernatants of expressing cells by the indicated antibodies are shown. The antigenic profiles of these two Envs are similar. **c** TM_mod_10 EKNA variants with modifications of the cleavage site, including a wild-type cleavage site (+) or a flexible linker ((GGS)_4_) replacing the cleavage site (modCS), were analyzed as in **a**. Note that the TM_mod_10 (+) EKNA Env is partially cleaved, as indicated by the presence of a gp120 band on SDS-PAGE. **d** Precipitation of the TM_mod_10 and TM_mod_10 EKNA variants by the indicated antibodies is shown. Data are representative of those obtained in at least two independent experiments

To determine if the secreted TM_mod_ Env(−)Δ712 glycoproteins oligomerize, the glycoproteins in the supernatants of expressing cells were analyzed on Blue Native gels. To evaluate the contribution of the transmembrane region to Env oligomerization, HIV-1_JR-FL_ sgp140(−) was included for comparison. The sgp140(−) glycoprotein is identical in sequence to the wild-type Env(−)Δ712 glycoprotein but is truncated at residue 684 and therefore lacks a transmembrane region. The secreted sgp140(−) glycoprotein formed trimers, dimers and monomers, which migrated on Blue Native gels more slowly than expected, as previously reported [48–52, 82, 83]. The secreted TM_mod_ Env(−)Δ712 glycoprotein mutants all exhibited similar overall patterns of migration on Blue Native gels (Figs. 2b, 3a and data not shown). Surprisingly, the TM_mod_ mutants, including TM_mod_10v2 and TM_mod_10v3, migrated as dimers (Figs. 2b, 3a and data not shown). Apparently, some alterations of the gp41 transmembrane region significantly affect the ability of the secreted Envs to form trimers. Given the similarity of the phenotypes of the TM_mod_ Env(−)Δ712 mutants, we selected TM_mod_10 for additional studies.

### Glycosylation of the TM_mod_10 glycoprotein

The wild-type HIV-1 Env is cotranslationally modified by a heavy coat of high-mannose carbohydrate chains [4, 8–10, 84]. After folding and trimerization, a small subset of surface-exposed glycans are converted in the Golgi apparatus to complex glycans [9, 10]. Envs with native compact structures typically are rich in high-mannose carbohydrates [8, 9, 55] and thus, are sensitive to deglycosylation by Endoglycosidase H. To evaluate glycosylation, the sgp140(−) and TM_mod_10 glycoproteins were treated with PNGase F, which removes all N-linked carbohydrates, and Endoglycosidase H_f_ (Endo H_f_), which removes only high-mannose carbohydrates. Both sgp140(−) and TM_mod_10 were resistant to Endo H_f_ digestion, indicating that they are extensively modified by complex carbohydrates (Fig. 2c). As a control, cells producing the TM_mod_10 glycoprotein were treated with kifunensine, a mannosidase I inhibitor [85]; the TM_mod_10 glycoprotein produced in kifunensine-treated cells was, as expected, efficiently deglycosylated by Endo H_f_ (Fig. 2d). Apparently, both the sgp140(−) and TM_mod_10 glycoproteins allow efficient access to glycosyltransferases in the Golgi apparatus that convert high-mannose carbohydrates to complex sugars.

### Antigenicity of the TM_mod_10 and TM_mod_10v2 mutants

The recognition of the sgp140(−), TM_mod_10 and TM_mod_10v2 glycoproteins by a panel of monoclonal antibodies was evaluated (Fig. 2e and f). The antigenicity of the three Env glycoproteins was similar. The 2G12 and VRC01 broadly neutralizing antibodies, which recognize a glycan-dependent epitope on the gp120 outer domain [86] and the CD4-binding site [87], respectively, precipitated the TM_mod_ Env(−)Δ712 mutants efficiently. These secreted Envs were also recognized by 19b, a weakly neutralizing antibody against the gp120 V3 region [88], and 902090, a poorly neutralizing antibody against a linear V2 epitope [89]. The 17b antibody, which recognizes a CD4-induced (CD4i) epitope that overlaps the CCR5/CXCR4-binding site on gp120, weakly precipitated the soluble Envs; the efficiency of 17b recognition was increased in the presence of sCD4, as expected [90]. These results indicate that some epitopes for weakly neutralizing antibodies are accessible on the TM_mod_ Env(−)Δ712 glycoproteins.

### Inclusion of gp41 cytoplasmic tail sequences in the TM_mod_ Env mutants

Sequences in the Env cytoplasmic tail have been suggested to influence the folding and/or conformation of the HIV-1 Env ectodomain [91–95]. We asked whether the addition of cytoplasmic tail sequences to the TM_mod_10 Env would affect the properties of the glycoprotein. Full-length, cleavage-negative HIV-1_JR-FL_ Env and Δ808 versions of TM_mod_10 were not secreted; a TM_mod_10 Env(−)Δ753 glycoprotein was secreted and, like the Δ712 glycoprotein, was mostly dimeric (Fig. 3a).

The PG9 and PG16 antibodies recognize quaternary epitopes in the gp120 trimer association domain [96–99]. Because the HIV-1_JR-FL_ Env is not recognized by these antibodies, we introduced the E168K + N188A (EKNA) changes into the TM_mod_10 Env(−)Δ712 and Env(−)Δ753 glycoproteins to restore the PG9 and PG16 epitopes. The TM_mod_ EKNA Env(−)Δ712 and Env(−)Δ753 glycoproteins were precipitated inefficiently by the PG9 and PG16 antibodies, less efficiently than by the VRC01, 2G12 or PGT121 antibodies (Fig. 3b). These results are consistent with the TM_mod_ EKNA Env(−)Δ712 and Env(−)Δ753 glycoproteins exhibiting a more open, less compact Env structure than the native Env.

### Modifications of the gp120-gp41 cleavage site

The proteolytic cleavage of gp120 and gp41 can influence the antigenicity of HIV-1 Env [76, 100–107]. We produced a version of TM_mod_10 EKNA called TM_mod_10(+) EKNA with a wild-type sequence at the gp120-gp41 cleavage site. Flexible linkers inserted at the gp120-gp41 cleavage site of sgp140 SOSIP.664 glycoproteins can mimic the effects of proteolytic cleavage on Env antigenicity [58, 59]. Therefore, we also produced a TM_mod_10 EKNA variant with a flexible linker at the gp120-gp41 cleavage site (TM_mod_10modCS EKNA). The migration of the secreted TM_mod_10 EKNA, TM_mod_10(+) EKNA and TM_mod_10modCS EKNA glycoproteins on Blue Native gels was similar, although the TM_mod_10(+) EKNA glycoprotein exhibited less distinct forms, possibly indicating greater heterogeneity (Fig. 3c). The antigenic profiles of these three glycoproteins were also similar (Fig. 3d). Thus, the modifications of the gp120-gp41 cleavage site had little effect on the oligomerization properties of the TM_mod_10 EKNA Env.

### Addition of C-terminal trimerization motifs to TM_mod_ Envs

**Fig. 4:**
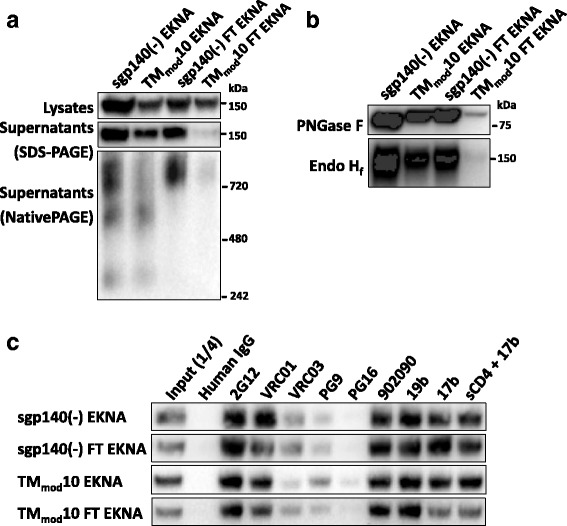
Effect of a fibritin trimerization motif on the TM_mod_10 Env. **a** Cell lysates and supernatants from 293T cells expressing the EKNA variants of sgp140(−) or TM_mod_10, or these Envs with a C-terminal fibritin trimerization domain (sgp140(−) FT and TM_mod_10 FT, respectively), were analyzed on gels and Western blotted. A polyclonal goat anti-gp120 antibody was used to detect the Envs on the Western blots shown in this figure. Addition of the fibritin domain to the C-terminus of TM_mod_10 did not prevent Env expression but significantly diminished release of the TM_mod_10 FT glycoprotein from the cells. **b** The indicated soluble Envs were subjected to digestion with PNGase F or Endo H_f_ and Western blotted. **c** Antibody precipitation of the EKNA variants of sgp140(−), sgp140(−) FT, TM_mod_10 and TM_mod_10 FT Envs secreted into the medium of expressing cells is shown. All EKNA Env constructs contain the E168K + N188A changes that restore the PG9/PG16 epitopes [96–99]. Data are representative of those obtained in duplicate or two independent experiments

The addition of C-terminal trimerization motifs from GCN4 or fibritin have been used to increase the homogeneity of soluble HIV-1 Env trimers [35, 44–47, 108]. As previously shown [44–47], the addition of a fibritin trimerization domain to the sgp140(−) glycoprotein resulted in efficiently secreted trimers (Fig. 4a). The TM_mod_10 FT glycoprotein with the fibritin motif was secreted inefficiently, but migrated on Blue Native gels in a manner consistent with a trimer. The TM_mod_10 FT glycoprotein was resistant to Endo H_f_, indicating that it is heavily modified by complex carbohydrates (Fig. 4b). Despite the greater relative homogeneity of the sgp140(−) FT and TM_mod_10 FT trimers, the antigenic profile of the EKNA variants of these glycoproteins was very similar to that of the sgp140(−) and TM_mod_10 Envs (Fig. 4c). Thus, all of the soluble Envs studied herein exhibit ectodomain conformations that are less compact than that of the native membrane Env.

### Stabilization of membrane Envs

**Fig. 5:**
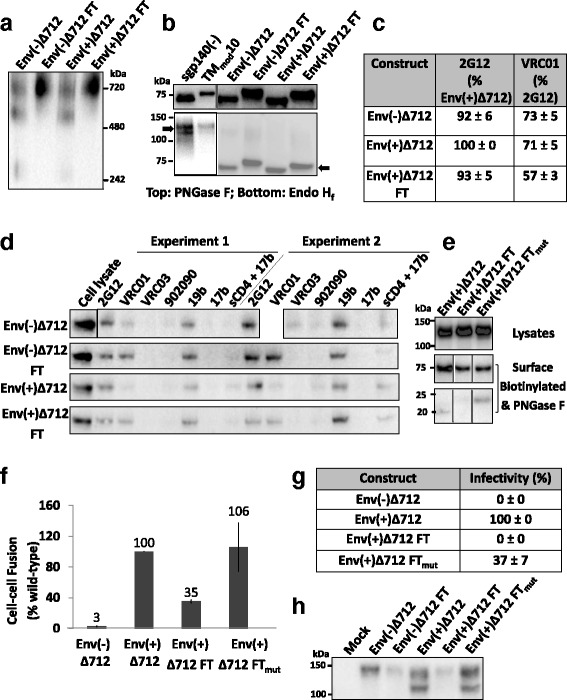
Effect of the C-terminal fibritin trimerization motif on membrane-anchored Envs. **a** Envs purified from transfected 293T cell membranes were analyzed by Blue Native PAGE. The Env(−)Δ712 FT and Env(+)Δ712 FT glycoproteins migrate at a size expected for trimers. **b** The purified soluble or membrane Envs were treated with PNGase F (*top panel*) or Endo H_f_ (*bottom panel*). The arrows indicate Envs after Endo H_f_ digestion. Compared to sgp140(−) and TM_mod_10 Envs, which are relatively resistant to Endo H_f_ digestion, purified membrane Envs are Endo H_f_-sensitive, and thus rich in high-mannose carbohydrates. **c** Flow cytometry was used to study Env surface expression and recognition by the 2G12 glycan-dependent antibody and the VRC01 CD4-binding site antibody. **d** To immunoprecipitate cell-surface Env, 293T cells transiently expressing the indicated Envs were incubated with antibodies, washed and lysed. Cell lysates were incubated with Protein A-Sepharose beads. Precipitates were Western blotted with a goat anti-gp120 antibody. **e** To assess cell-surface Env processing, cells expressing the indicated Envs were biotinylated as described in Methods. The cell lysates (*upper panel*) or deglycosylated cell-surface Envs (*lower panels*) were Western blotted with the 4E10 anti-gp41 antibody. After deglycosylation, the uncleaved Env is 75 kD, and the cleaved transmembrane Envs are 20–23 kD. **f** An α-complementation assay was used to measure Env-mediated cell-cell fusion. The reduced cell-cell fusion activity of Env(+)Δ712 FT was restored by the disruption of fibritin trimerization in the Env(+)Δ712 FT_mut_ glycoprotein. **g** The infectivity of recombinant luciferase-expressing HIV-1 with the indicated Envs was measured on Cf2Th-CD4/CCR5 target cells. The luciferase activity in the target cells was normalized to that observed for the Env(+)Δ712 glycoprotein. **h** Virions pseudotyped with the indicated Env proteins were purified through a 20% sucrose cushion, denatured and Western blotted. Data in this figure are representative of, or averaged from, those obtained in at least two independent experiments. Error bars are standard deviations

Membrane-anchored HIV-1 Envs represent native, functional Env forms, but are not as efficiently expressed or as conveniently purified as soluble Envs. Moreover, after detergent solubilization of Env from membranes, Env trimers typically exhibit instability [109–111]. We investigated the value of adding a C-terminal fibritin trimerization domain to the HIV-1_JR-FL_ Env(+)Δ712 and Env(−)Δ712 glycoproteins. These membrane-anchored, cytoplasmic tail-deleted Envs are identical, except that the gp120-gp41 cleavage site is altered in the latter construct. The Env(+)Δ712 and Env(−)Δ712 glycoproteins and their counterparts with C-terminal fibritin trimerization domains were expressed transiently in 293T cells. Membranes were prepared from the cells and the membrane Envs were solubilized in detergents. The detergent solutions were immediately analyzed on Blue Native gels, and the Envs were detected by Western blotting. In addition to the trimeric forms, nearly half of the Env(−)Δ712 and Env(+)Δ712 glycoproteins (without the C-terminal fibritin trimerization domain) migrated as dimers, and another smaller fraction migrated as monomers (Fig. 5a). By contrast, the Env(−)Δ712 FT and Env(+)Δ712 FT glycoproteins migrated predominantly as trimers. Thus, the trimeric forms of the Env(−)Δ712 FT and Env(+)Δ712 FT glycoproteins are stabilized by the addition of a C-terminal fibritin trimerization domain.

The glycosylation of the Env(−)Δ712, Env(+)Δ712, Env(−)Δ712 FT and Env(+)Δ712 FT glycoproteins purified from membranes was examined. All four glycoproteins were efficiently deglycosylated by Endo H_f_, in contrast to the sgp140(−) and TM_mod_10 glycoproteins (Fig. 5b). Thus, as expected for native membrane Envs, the Env(−)Δ712, Env(−)Δ712 FT, Env(+)Δ712 and Env(+)Δ712 FT glycoproteins are mainly modified by high-mannose carbohydrates. This property does not apparently change with the addition of the C-terminal fibritin motif.

The Env(+)Δ712 FT glycoprotein was efficiently expressed on the surface of transfected 293T cells (Fig. 5c). Unlike the soluble Envs (Fig. 4c), the membrane-anchored Envs were inefficiently recognized by the poorly neutralizing antibodies 902090 and 17b (Fig. 5d). Following incubation with soluble CD4 (sCD4), 17b recognition of the Envs was increased. The proteolytic processing of the Env(+)Δ712 FT glycoprotein was less efficient than that of the Env(+)Δ712 glycoprotein (Fig. 5e). We tested the ability of the HIV-1_JR-FL_ Env(+)Δ712, Env(+)Δ712 FT and Env(−)Δ712 FT glycoproteins to mediate cell-cell fusion in an α-complementation assay. The Env(+)Δ712 glycoprotein mediated cell-cell fusion very efficiently in this assay (Fig. 5f). Consistent with the importance of gp120-gp41 cleavage for HIV-1 Env function, the activity of the Env(−)Δ712 glycoprotein was near the background level of the assay. The Env(+)Δ712 FT glycoprotein mediated cell-cell fusion at 35% of the efficiency of the Env(+)Δ712 glycoprotein, consistent with the lower degree of processing of Env(+)Δ712 FT (Fig. 5e). We tested the hypothesis that the interactions among the fibritin trimerization domains at the C-terminus of the Env(+)Δ712 FT glycoproteins contributed to the relative decrease in the processing and syncytium-forming ability of this Env variant. Consistent with this hypothesis, the Env(+)Δ712 FT_mut_ glycoprotein, in which the residues Y469 and R471 that contribute to trimerization of the fibritin domain were altered, mediated cell-cell fusion as efficiently as the Env(+)Δ712 glycoprotein (Fig. 5f). The Env(+)Δ712 FT_mut_ glycoprotein was cleaved more efficiently than the Env(+)Δ712 FT glycoprotein (Fig. 5e), providing one explanation for the better syncytium-forming ability of Env(+)Δ712 FT_mut._

The ability of the membrane Env variants to mediate virus entry was evaluated in a single-round *env* complementation assay. The HIV-1_JR-FL_ Env(+)Δ712 FT glycoprotein did not detectably support virus entry into Cf2Th-CD4/CCR5 cells in this assay, whereas the Env(+)Δ712 FT_mut_ glycoprotein mediated infection at a level 37% of that observed for the Env(+)Δ712 glycoprotein (Fig. 5g). Differences in the amount of mature, processed Env glycoproteins in the virions correlated with the differences in virus infection (Fig. 5h). Taken together, these results suggest that the presence of a fibritin trimerization domain at the C-terminus of the Env(+)Δ712 glycoprotein can affects its incorporation into virions.

## Discussion

CD4 binding has been shown to trigger transformation of the HIV-1 Env from an unliganded conformation (State 1) to an intermediate conformation (State 2) and then to the prehairpin intermediate (State 3) [13, 14]. The degree to which HIV-1 Env variants sample these conformational states spontaneously, in the absence of CD4, can determine their ability to utilize low levels of target cell CD4 and their susceptibility to antibody neutralization [12, 14]. Therefore, primary HIV-1 Envs need to maintain Env in a State 1 conformation, a situation that depends upon the integrity of multiple gp120 and gp41 components, including the membrane-interactive elements of gp41, i.e., the transmembrane region and MPER [12, 14, 71–74]. We investigated various strategies for expressing HIV-1 Envs that contain minimally modified ectodomains and include membrane-interactive components.

Guided by the available information, we altered the helical faces of the transmembrane coiled coil that are predicted to interact with the lipid bilayer [62, 66]. The wild-type HIV-1 Env has leucine, valine and isoleucine residues on these helical faces; this is consistent with the preference for these hydrophobic amino acids at the lipid interface in many multispanning alpha-helical membrane proteins [112]. Substitution of single alanine residues in this putative lipid-interactive surface of the transmembrane region exerted no discernible effect on membrane anchorage. However, when combined charged, polar and alanine residues replaced the six hydrophobic residues naturally located on these helical faces, the Env complex was secreted into the medium. A control Env mutant in which the residues predicted to face the interior of the transmembrane coiled coil were replaced by charged residues expressed only at low steady-state levels and may have been poorly folded. Thus, we disrupted a stop-transfer signal in the Env transmembrane coiled coil by altering predicted lipid-interactive faces of the HIV-1 transmembrane helical coiled coil. The membrane-anchorage phenotypes of the Env mutant panel support the orientation of the Env transmembrane helices predicted by the structural models [62, 66].

As previously observed [34–40], soluble cleavage-negative sgp140(−) Envs lacking the transmembrane anchor formed heterogeneous oligomers, including trimers [44]. The addition of the Env transmembrane region, engineered to remove stop-transfer signals, significantly increased the percentage of secreted Envs in dimeric forms. Apparently, the interprotomer interactions that are predicted to occur among the transmembrane helices in the wild-type Env are not maintained efficiently in the secreted TM_mod_ Envs. The favorable energetics of such interactions in a membrane environment may not apply in the context of a soluble protein. Although the formation of soluble Env trimers could be restored by the addition of a known trimerization domain from T4 bacteriophage fibritin [80], the resulting trimers were similar in conformation to comparable soluble Env constructs lacking the gp41 transmembrane region. The significant amount of complex carbohydrates and the antigenic profile of both the soluble gp140 and TM_mod_ Env constructs, regardless of the addition of the fibritin motif, indicate significant differences in conformation from that of native HIV-1 Envs. The absence of the interaction of the gp41 transmembrane region and/or MPER with the membrane may result in an increase in the overall conformational flexibility of the Env protomers in these soluble glycoprotein trimers. Indeed, changes in gp41 transmembrane residues and MPER residues near the viral membrane have been shown to disrupt the maintenance of the HIV-1 Env in a State 1 conformation [12, 71–74]. Thus, achieving a topology of the gp41 transmembrane region that supports a native State 1 Env conformation in the absence of a membrane-like environment remains an elusive goal.

Certain conformations of the HIV-1 Env trimer ectodomains have been stabilized for structural analysis by the introduction of gp120 and gp41 changes, or selected by the binding of specific antibodies [48–59]. In all of these structures, the gp41 membrane-spanning region and MPER are disordered, leaving open the possibility that the interaction with the membrane may make important contributions to the maintenance of Env structures other than those stabilized states. A complete understanding of the conformations sampled by the native unliganded and receptor-bound HIV-1 Env may thus require the study of Envs in membrane-anchored contexts. To this end, we compared the properties of uncleaved and cleaved Envs anchored in membranes, specifically demonstrating that the addition of a fibritin trimerization domain to the C-terminus could help to maintain trimer stability after Env solubilization in detergent solutions. In contrast to the soluble Envs, the membrane Envs exhibited a predominantly high-mannose carbohydrate profile, indicative of a compact ectodomain with restricted accessibility of surface glycans.

The Env(+)Δ712 FT glycoprotein was expressed on the surface of cells nearly as efficiently as its Env(+)Δ712 counterpart, which lacks the fibritin trimerization (FT) domain. Of interest, the proteolytic processing efficiency and cell-cell fusing capacity of the Env(+)Δ712 FT glycoprotein were lower than that of the Env(+)Δ712 glycoprotein. This decreased activity of Env(+)Δ712 FT was relieved by alteration of the trimer interface of the FT motif, suggesting that the oligomeric interactions of the C-terminal fibritin domain limit gp120-gp41 cleavage and the syncytium-forming ability of Env in this context. Albeit decreased compared with the Env(+)Δ712 glycoprotein, the intact cell-cell fusing activity of the Env(+)Δ712 FT glycoprotein and the observed increase in the stability of these trimers makes them attractive candidates for further study. For example, these trimers could be useful tools to investigate the impact of Env cytoplasmic components on ectodomain conformation and function.

The trimerization of the C-terminal fibritin domain on the Env(+)Δ712 FT glycoprotein appears to interfere with the ability of the pseudotyped virions to support infection. Compared with the Env(+)Δ712 and Env(−)Δ712 counterparts, the respective Env(+)Δ712 FT and Env(−)Δ712 FT glycoproteins were incorporated less efficiently into virion particles. Even more striking was the relative reduction in the amount of the proteolytically cleaved Env in virions for Env(+)Δ712 FT compared with Env(+)Δ712. These phenotypes were partially reversed by alteration of the residues implicated in trimerization of the C-terminal fibritin motif. These results suggest that the presence of a trimeric fibritin domain in the Env cytoplasmic tail may have interfered with the efficient incorporation of Env, particularly the mature Env, into HIV-1 virions.

The studies reported herein underscore the difficulty of mimicking the functional State 1 conformation of Env, which represents a major target for many small-molecule entry inhibitors and most broadly neutralizing antibodies [13, 14, 28], with soluble Envs. Our work provides a path toward purification and characterization of HIV-1 Envs that are anchored in the membrane and retain the ability to fuse membranes. Such studies may be critical to the discovery and optimization of interventions that disrupt the process of virus entry for the purposes of HIV-1 treatment and prophylaxis.

## Conclusions

Introduction of at least two polar/charged residues into the proposed lipid-facing side of the gp41 transmembrane helical coiled coil resulted in release of the HIV-1 Env from the membrane. These soluble TM_mod_ Envs were dimeric and loosely associated, implying that the modified transmembrane region minimally contributes to the trimerization and antigenicity of the soluble Envs.

In contrast to the soluble Envs, the membrane-bound Env assumed a compact conformation and thus is of interest for structural and vaccine studies. Addition of the C-terminal fibritin motif stabilized the membrane Env trimer during preparation and represents a promising strategy for purification and characterization of native-like Envs.

## Abbreviations

Cryo-EM: Cryo-electron microscopy
CS: Cleavage site
DTT: dithiothreitol
EKNA: E168K/N188A
Endo H_f_: Endoglycosidase H_f_
Env: Envelope glycoprotein
ER: Endoplasmic reticulum
FBS: Fetal bovine serum
FT: Fibritin
HIV-1: Human Immunodeficiency Virus Type 1
HR: Heptad repeat region
HRP: Horseradish peroxidase
MPER: Membrane-proximal external region
NMR: Nuclear magnetic resonance
sCD4: Soluble CD4
SDS-PAGE: Sodium dodecyl sulfate-polyacrylamide gel electrophoresis
TM: Transmembrane region.
